# Fourier imaging for nanophotonics

**DOI:** 10.1515/nanoph-2023-0887

**Published:** 2024-02-27

**Authors:** Sébastien Cueff, Lotfi Berguiga, Hai Son Nguyen

**Affiliations:** Univ Lyon, CNRS, ECL, INSA Lyon, UCBL, CPE, INL UMR5270, 69134 Ecully, France; CNRS, Ecole Centrale de Lyon, INSA Lyon, Universite Claude Bernard Lyon 1, CPE Lyon, INL, UMR5270, 69134 Ecully, France

**Keywords:** Fourier optics, light emitters, Mie resonances, metasurfaces, spectroscopy, imaging

## Abstract

Standard optical characterization and spectroscopy techniques rely on the measurement of specular reflection, transmission, or emission at normal incidence. Although the usefulness of these methods is without question, they do not provide information on the angular dependence of the scattered light and, therefore, miss crucial insights on the physical processes governing light emission and scattering. In this Review, we explain the basics of Fourier imaging and show how it can be used to measure the angular distribution of scattered light in single-shot measurements. We then give a comprehensive panorama on recent research exploiting this technique to analyze nanostructures and detail how it unlocks fundamental understandings on the underlying physics of nanophotonic structures. We finally describe how simple additions to a Fourier imaging setup enable measuring not only the radiation pattern of an object but also the energy, polarization, and phase toward resolving all aspects of light in real time.

## 1 Introduction

In the last decades, the advances in nanofabrication techniques have led to the ubiquitous use of nanomaterials and nanostructures in a myriad of applications ranging from quantum optics, spectroscopy, imaging, and new generation lighting systems [1–10]. This progress was accompanied, if not driven, by a continuous development of analytical measurement and inspection techniques, which most often depend on optical methods. One of the most well-known examples of such methods is spectroscopic ellipsometry, which played a major role in the development of integrated-circuit technology by providing nondestructive measurements of materials assembled at the micro- to nanoscale [11], [12]. Similarly, optical spectroscopy has been a key technique to analyze and understand the fundamentals of light emission and absorption processes [13]–[15]. These two pervasive techniques share similarities: they both measure the energy spectrum of light emitted from or reflected off a sample with no or little attention paid to the angle into which light is radiated from the object under study. In standard ellipsometry, one only measures light that has been reflected off in specular directions; hence, all of the scattered light emerging at nonspecular angles are discarded. On the contrary, optical spectroscopy collects light emitted from a distribution of angles using high numerical aperture objectives, but all angles are summed up at the detection stage and one cannot resolve the preferred directions of emission. In other words, these techniques dismiss the angular distribution of light. Recent advances in nanophotonics call for more advanced measurement methods to precisely characterize the fundamental properties of nano-objects and devices. In particular, the multipolar character of engineered nanostructures as well as the collective effects in metasurfaces provide a wealth of possibilities in terms of scattering directionality that cannot be resolved easily with standard optical measurement techniques.

While there are straightforward approaches in obtaining the angular distribution of scattered light, by mechanically scanning sources and detectors (using e.g., gonioreflectometers for scatterometry or bidirectional reflectance distribution [16–20]), these methods are slow, cumbersome, and most importantly preclude real-time measurements and, therefore, cannot capture the time dynamics of the angular distribution of light.

Our goal in this Review is to show how Fourier imaging techniques provide a strikingly simple way to measure the angular distribution of light scattering: most of the time, one simply needs to add a standard lens in their optical setups. We further detail how the addition of angular resolution to optical measurements unlocks access to a wealth of information on nanostructures that are overlooked by standard spectroscopic techniques. We then take advantage of this opportunity to describe recent works on nano-objects and metasurfaces as well as to explain the role played by Fourier imaging to push forward the fundamental understanding in the field of nanophotonics.

We note that several different terminologies exist in the literature to design Fourier optics-based techniques to resolve the angular distribution of light, e.g., Back-Focal Plane imaging, momentum-resolved optical characterization, energy and momentum spectroscopy, k-space imaging, and Fourier microscopy. We decided for this review to encompass all of these techniques under the general term Fourier Imaging.

The Review is organized as follows: in Section 2, we provide a concise description of the physics of Fourier optics starting with the well-known Fourier transform properties of a lens and describe how lenses can be used to measure the angular distribution of light. Section 3 describes recently measured radiation patterns from various nano-objects including emitters, single scatterers, and emitters-nanostructure systems. Next, in Section 4, we review the use of Fourier imaging in more complex setups with not only radiation patterns measurements but also energy, polarization, and phase toward spectroscopy techniques resolving all aspects of light. Finally, in Section 5, we discuss the perspectives and future prospects of this technique.

## 2 Fundamentals of Fourier imaging

Very often, signals that carry physical information (electrical, acoustic, optical, …) vary over time and are distorted along the way, either during propagation or at the detection step. As Fourier transformation enables decomposing time-varying signals into their frequency components, Fourier analysis is a universal tool for scientists, especially in the fields of communications and electrical engineering. Among the countless possibilities offered by this technique, it is exploited as a flexible way to analyze the harmonics of periodic signals, to synthesize radiowave antenna patterns, or to filter out detected signals and extract meaningful information out of them.

Why should we care about Fourier analysis in the field of nanophotonics? First of all, because of what we could consider as a happy accident of optics: a simple lens naturally “computes” the Fourier transform of a given light field. How can that be possible? The answer to this question goes back centuries, when light was starting to be understood as a wave. The successive analysis of light scattering phenomena from different objects by Huygens, Young, and Fresnel lead to the emergence of diffraction theory. The so-called Huygens–Fresnel principle enables calculating the electromagnetic fields resulting from the diffraction of a subwavelength sized object at an arbitrary distance from it. Under the Fraunhofer approximation (i.e., in the far-field), the Huygens–Fresnel principle takes the mathematical form of a 2D Fourier transform (up to multiplicative phase factor). The light propagation of the diffracted pattern behaves as a short pass filter and at infinity as a Fourier transform.

In the case of a converging lens, an incident plane wave is converted by the lens to a spherical wave with radius *f* converging toward the image focal point. This is the original function of a lens: light from an object at infinity focuses at the focal point, forming a reduced or magnified pattern of the source. Such an operation on incoming fields mathematically translates into a Fourier transform. The field *E*(*u*, *v*) with spatial frequencies $f_{x}=\frac{u}{\lambda f}$ and $f_{y}=\frac{v}{\lambda f}$ , at the focal plane, originating from a field *E*(*x*, *y*) at a distance *d* before the lens can be written as [21]:

(1) $$E\left(f_{x},f_{y}\right)=\frac{e^{\mathrm{ikf}}}{i\lambda f}e^{i\pi \lambda f\left(1-\frac{d}{f}\right)\left(f_{x}^{2}+f_{y}^{2}\right)}F_{f_{x},f_{y}}\left[E\left(x,y\right)\right],$$

The wavefront at the focal plane is the Fourier transform of the field distribution in the plane located at distance *d* from the lens, with an additional phase term. Interestingly, this phase term vanishes when the object under study is located at the object focal plane, i.e., *d* = *f*, the former equation (1) becomes (with (*u*, *v*) the spatial coordinates in the focal plane):

(2) $$E\left(u,v\right)=\frac{e^{\mathrm{ikf}}}{i\lambda f}F_{f_{x},f_{y}}\left[E\left(x,y\right)\right]$$

(3) $$E\left(u,v\right)=\frac{e^{\frac{2i\pi f}{\lambda }}}{i\lambda f}\underset{x}{\int }\underset{y}{\int }E\left(x,y\right)e^{-\frac{2i\pi }{\lambda f}\left(ux+vy\right)}dxdy$$

When expressed in terms of spatial frequencies $f_{x}=\frac{u}{\lambda f}$ and $f_{y}=\frac{v}{\lambda f}$ this field *E*(*u*, *v*) at the back focal plane (BFP) is, therefore, equivalent to the Fourier transform of the incident field (up to a constant multiplicative term). This is the reason why the back focal plane is also named the *Fourier plane*.

To illustrate how this operation can be exploited in various characterization schemes, let us consider the case of a single plane wave with amplitude *E*_0_ leaving the object plane with a specific angle, characterized by a wavevector **k** = (*k*_*x*_, *k*_*y*_, *k*_*z*_) with $k_{x}=\frac{2\pi u_{0}}{\lambda f}$ and $k_{y}=\frac{2\pi v_{0}}{\lambda f}$ . Using the expression above, the field at the BFP becomes

(4) $$\begin{array}{ll} E\left(u,v\right) & =\frac{e^{\frac{2i\pi f}{\lambda }}}{i\lambda f}\underset{x}{\int }\underset{y}{\int }E_{0}e^{-i\left(k_{x}x+k_{y}y\right)}e^{-\frac{2i\pi }{\lambda f}\left(ux+uy\right)}dxdy\\ & =\frac{e^{\frac{2i\pi f}{\lambda }}}{i\lambda f}E_{0}\delta \left(u+u_{0},v+v_{0}\right). \end{array}$$

**Figure 1:**
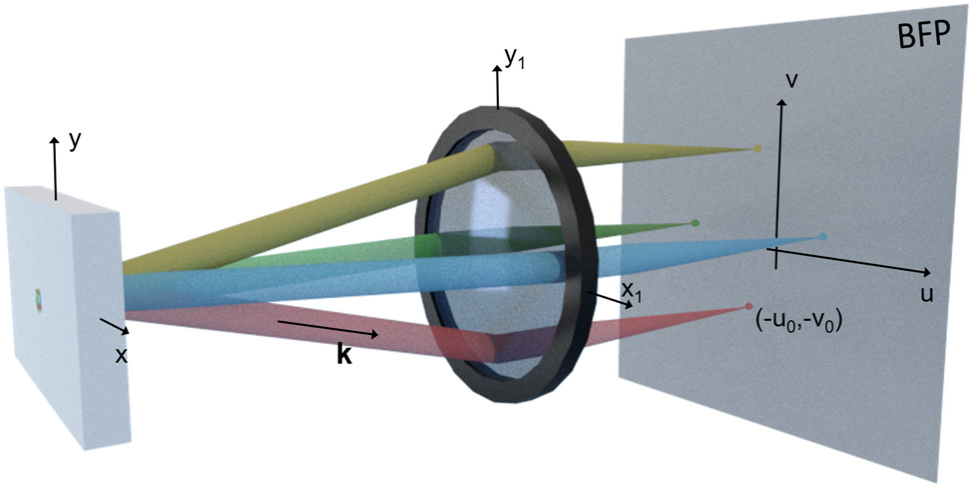
Artist view illustrating the principle of Fourier imaging. Radiation from a source is depicted as a superposition of plane waves (shown in different colors), each of which is focused at a different position in the back focal plane to produce an image representing the radiation pattern of the source.

We can immediately see that this plane wave is converted into a spherical wave whose focus is shifted from the optical axis (see Figure 1) and located at the position (−*u*_0_, − *v*_0_) in the BFP.

Let us now consider the more realistic case of an arbitrary source, comprising a sum of plane waves that are described by their respective **k** wavevectors. As each wavevector corresponds to a propagation direction, each of them will respectively focus at one specific point of the BFP, forming an image that represents the angular distribution of the input field. Or to put it another way, by imaging the BFP, one can directly measure the radiation pattern of the source. From this radiation pattern, it is straightforward to calculate the different wave vector components of the source, provided the apodization function of the lens (or objective) is known. In most cases (for example, the M Plan Apo objectives), they obey the Abbe sine condition; hence, the relation between the radial position *r* at the BFP and the angle of incidence *θ* is *r* = *f* sin(*θ*). It leads to the following relations between the position in the BFP of the objective lens and the wavevector **k** = (*k*_*x*_, *k*_*y*_, *k*_*z*_):

(5) $$\begin{array}{cc} k_{x} & =\frac{2\pi }{\lambda }n\sin\theta \cos\phi =\frac{2\pi }{\lambda }\frac{r\cos\phi }{f}\\ k_{y} & =\frac{2\pi }{\lambda }n\sin\theta \sin\phi =\frac{2\pi }{\lambda }\frac{r\sin\phi }{f} \end{array}$$

with *ϕ* the angular position in the BFP. This example illustrates the analogy with “conventional” Fourier transform operations, in which we decompose time-varying signals into their temporal frequency components. We take advantage of this analogy to emphasize that the principle exploited in Fourier-Transform Infrared (FTIR) spectroscopy should not be confused with the Fourier imaging techniques described in this manuscript. Indeed, in a FTIR equipment, an interferometer with a moving mirror produces a time-modulated light beam that interacts with a sample [22]. The resulting interferogram is detected as a function of time at the output of the sample and postprocessed using Fast Fourier-Transform algorithms to obtain a frequency (wavenumber) spectrum. FTIR, therefore, leverages *time-frequency* transformations performed with a computer. On the contrary, in the case of Fourier Optics, a lens decomposes an optical *wavefront* into its *spatial frequencies*. The two techniques, therefore, share a similar formalism but are radically different in their use and applications.

In the former equation, we did not take into account the aperture of the lens (hence, the Numerical aperture) that limits the space field and so changes the Dirac function by an Airy profile. It is worth noting that the Fourier transform is available beyond the paraxial approximation for numerical aperture, up to 0.7 [23]. Even for very high numerical aperture, vectorial Debye integral should be used to describe the vectorial nature of the field after lens transformation [23]. Nevertheless, the lens transformation can still be related to a Fourier transform as used for the numerical computation [24], [25].

If we summarize what we have learned in this section, an arbitrary wavefront originating from the front focal plane of a lens will form an image at the BFP that represents its angular distribution. Each position in the BFP, therefore, corresponds to a plane wave, characterized by a wavevector **k**. Imaging this wavevector distribution, therefore, allows measuring the radiation pattern of an arbitrary source.

Building an optical setup to image the BFP of an objective is not a difficult task but may require careful designs depending on the needs and existing setups. As these considerations are out of the scope of the present review, we redirect the readers to the work of Kurvits et al. that provides a detailed and extensive study on the different possible objectives and configurations for Fourier imaging as well as their respective pros and cons [26].

## 3 Measuring and analyzing radiation patterns

The most straightforward use of Fourier imaging is to measure the angular distribution of light arising from an object. In this section, we review how this principle can be exploited to analyze and understand light emission and scattering from a broad diversity of nano-objects.

### 3.1 Light emitters

Spontaneous light emission and scattering of light by small objects have been investigated for decades and bear strong similarities – in both cases, photons are emitted as a result of accelerated electric charges. There is, however, a notable difference: while the spontaneous emission characteristics are set by the quantum-mechanical properties of the emitter, light scattering is governed by the dimension and geometry of the scatterer. While a standard method of characterizing emitters and scatterers is to collect their light spectra, analyzing their radiation patterns provide crucial insights in the physics governing their properties.

**Figure 2:**
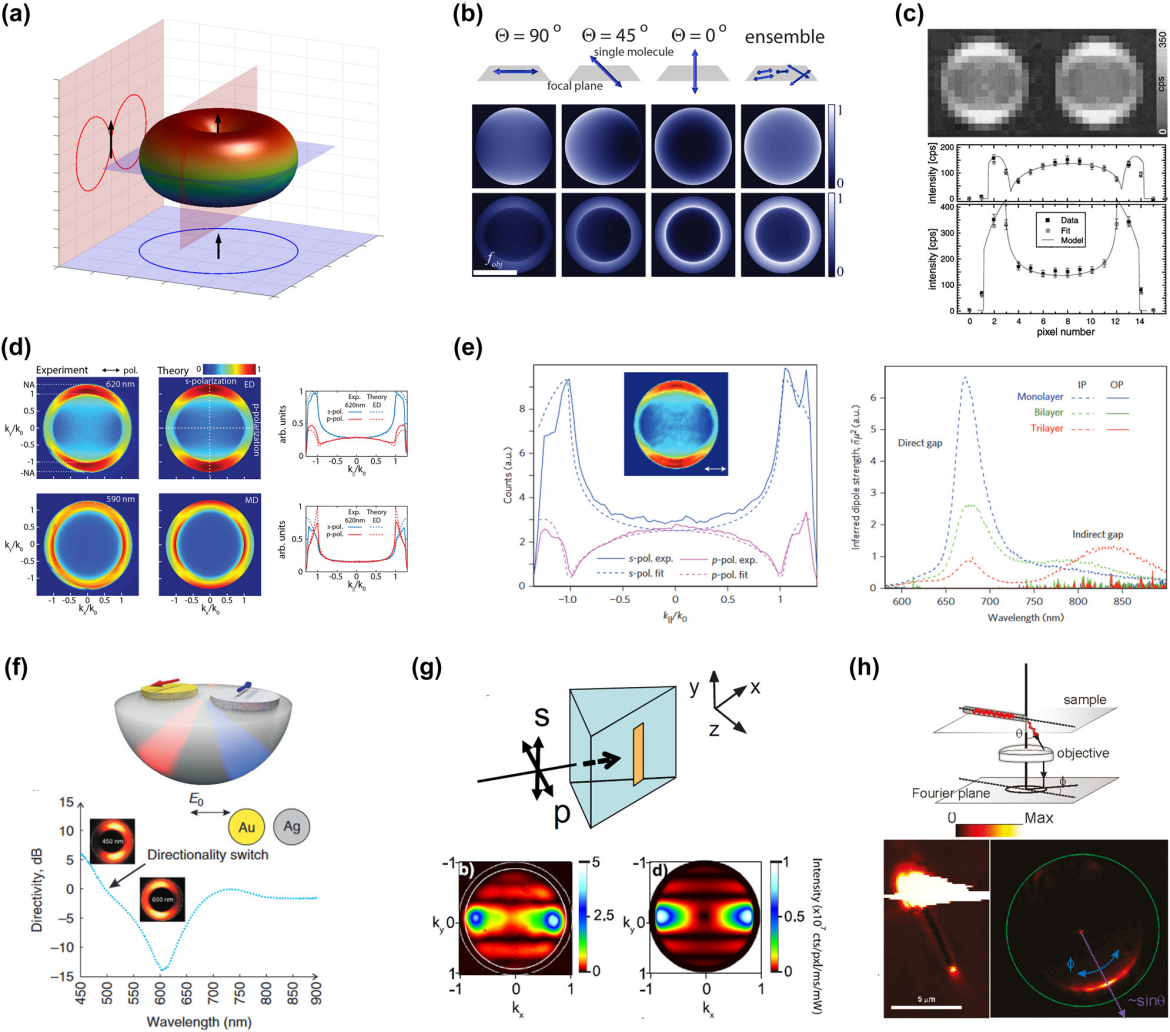
Radiation patterns and angular-distribution of light emitters and scatterers measured by Fourier imaging. (a) A dipolar source typically radiates light in a doughnut pattern with a sine square cross section. (b) The image collected at the back-focal plane of a lens or objective will be strongly affected by the orientation of the dipole with respect to the focal plane (figure inspired by the work from Backer et al. [28]). (c) Lieb et al. were the first to experimentally measure the radiation patterns of single emitters at the BFP of an objective [29]. (d) By leveraging self-interference effects in thin films, Taminiau et al. succeeded to untangle electric dipole and magnetic dipole contributions to light emission from Europium ions [30]. (e) Conversely, Schuller et al. resolved the orientation of dipoles within few-layers MoS_2_ [35]. (f) Bi-metallic nanoantenna dimers enabling color routing [46]. (g) Plasmonic nanoantenna excited in a total internal reflection configuration, the measured BFP imaging shows a sinc^2^ dependence, in agreement with the theory [47]. (h) Directional scattering from a silver nanowire resolved using BFP imaging [48]. (c) Reproduced with permission [29]. Copyright 2004, Optica Publishing Group. (d) Reproduced with permission [30]. Copyright 2012, Nature Publishing Group. (e) Reproduced with permission [35]. Copyright 2013, Nature Publishing Group. (f) Reproduced with permission [46]. Copyright 2011, Nature Publishing Group. (g) Reproduced with permission [47]. Copyright 2011, IOPScience. (h) Reproduced with permission [48]. Copyright 2011, American Chemical Society.

In the most simplified case, light emission and scattering can be considered as originating from a point-dipole source: an infinitesimal charge oscillating in time [6], [27]. The resulting angular-distribution of emitted photons is not isotropic but rather take on a typical sine square radiation pattern that is sometimes referred to as a “doughnut pattern,” as displayed in Figure 2(a). Given the symmetries of such a pattern, the collected light will, therefore, strongly depend on the respective positions of the detection system and the orientation of the dipole. As depicted in Figure 2(b), this results in distinct patterns collected in the BFP of an objective for different orientations of dipoles with respect to the plane of incidence [28]. A pioneering work of Lieb et al. [29] exploited this effect to directly resolve the orientations of single molecules by measuring their radiation patterns in the BFP of an objective (Figure 2c). This method enables mapping the orientations of single molecules but cannot be used for ensembles of emitters [28]. Indeed, as shown in Figure (2b), the contributions of isotropically oriented emitters average out in the far field, resulting in a symmetric angular distribution in the Fourier plane. Taminiau et al., however, provided an elegant method to gain insights on the physics of light emission from an ensemble of emitters [30]. By exploiting self-interference effects at the emitters’ location in a thin film, they were able to untangle the contributions of electric dipoles (ED) and magnetic dipoles (MD) from trivalent Europium ions. This was made possible because of the different symmetries in the radiation patterns of ED and MD, resulting in distinct angular distribution in the BFP, as displayed in Figure 2d). The same method was then used to quantify magnetic dipole contributions to light emission in erbium [31], [32] and various other rare-earth emitters [33], as well as in 2D lead halide perovskites [34]. The self-interference effect in thin films was exploited by the same group to resolve the orientation of electric dipoles in mono-, bi-, and tri-layer MoS_2_, revealing that light emission from this layered material solely originates from in-plane dipoles, irrespective of the number of layers [35]. Later on, this principle was used to determine the dipole orientation in organic light-emitting diodes [36], in indium selenide [37], in van der Waals heterostructures [38], [39], as well as to control the tailored dipole orientations of CdSe nanoplatelets [40], [41]. Conversely, combining Fourier imaging with absorption measurements enabled resolving the optical anisotropies of polymers [42], [43] and layered perovskites [44], as well as revealing a nonzero natural magnetic polarizability in a layered perovskite [45]. Fourier imaging, therefore, enabled significant fundamental insights into the physics of light emission within single and ensemble of emitters.

### 3.2 Light scatterers

In the previous part, we understood how Fourier imaging provides a powerful means to obtain fundamental insights on the nature and orientation of emitting dipoles. Such a concept can definitely find a resonance for the study of light scattering by nano-objects. Consider a collection of atoms assembled into an arbitrary object. Upon excitation with an electromagnetic field, induced dipoles within the object oscillate at the frequency of the applied field and scatter light in all directions. The total scattered light measured in the far field is the result of the superposition of all secondary waves produced by the induced radiating dipoles within the object. As this scattering is coherent, secondary waves may interfere constructively or destructively depending on the phase differences for a given direction. The radiation pattern is, therefore, governed both by the size, shape, and nature of the object, or in other words, by the particle’s complex polarizability.

A simple example of the size dependence is the scattering by spheres that support Mie resonances. Consider a spherical particle with a constant refractive index and a size parameter $x=\frac{2\pi nR}{\lambda }$ , where *n* is the refractive index of the surrounding medium, *λ* the wavelength of the incident light in vacuum, and *R* the sphere’s radius. When the size parameter *x* increases from 1 to 20, the forward scattering is strongly enhanced while the backward scattering is suppressed [49]. Engineering the size, shape, and nature of objects may, therefore, give rise to endless possibilities of radiation patterns.

With this principle in mind, recent progress in nanotechnology has allowed researchers to shape matter at the nanoscale, so as to engineer objects with precise optical scattering properties. These structures may be fabricated from metallic or high-index dielectric materials and can be designed to support strong optical resonances as well as antenna-like properties that can be leveraged to tailor the amplitude, momentum, phase, and polarization of electromagnetic fields. Such subwavelength objects have been exploited to design nanophotonic devices that locally confine, enhance, and mold electromagnetic fields for various applications such as sensing [50]–[53], light detection [54], imaging [55]–[57], and structural coloration [58], [59].

The wealth of possibilities offered by such building blocks to mold optical fields and radiation patterns call for advanced methods to thoroughly characterize their intrinsic properties. Thus, it becomes crucial to inspect and analyze the scattering properties of these optical resonators as fabricated, and for this, measuring the angle-resolved radiation pattern is necessary.

One of the simplest nano-object that scatters light at optical frequencies is a metallic nanoparticle supporting plasmonic resonances. Measuring the radiation patterns of such scatterers is not an easy task. Contrarily to the case of light emitters, a practical problem often arises in the analysis of radiation patterns from these resonators: incident and scattered fields overlap on the wavelength axis. It is, therefore, challenging to only detect the scattered signal and block the much stronger illumination signal. Huang et al. [60] used a beam stop in the BFP to filter out the illumination signal while retaining the contribution from the analyzed gold nanoparticles above critical angles. While this detected signal only represents a small portion of the total BFP, by imaging large wavenumbers of the BFP, they were able to resolve radiation patterns of monomers and dimers of Au nanoparticles and experimentally measured an angular distribution of light showing a sine squared angular dependence. This experiment demonstrates these nanoparticles typically radiate as point-dipoles, in which the majority of light is radiated perpendicular to the dipole moment, hence, bearing strong similarities with single emitters.

The nature of nanoparticles also plays an important role in the scattering properties. For example, the differences in permittivity between gold and silver makes their plasmon resonances occur at separate wavelengths. By fabricating closely spaced Ag–Au nanoantennas, Shegai et al. [46] showed that the phase shift between the two resonances result in constructive interferences in the red for one direction and constructive interferences in the blue for a different direction. Using Fourier imaging, they directly showed the different scattering directions for blue and red wavelengths and, hence, demonstrated a directional color routing (see Figure 2f).

Keeping a similar geometry – spherical nanoparticles – but using instead a dielectric material with high refractive index expands the possibilities for scattering. For example, it has been shown that spherical silicon nanoparticles can host a variety of Mie resonances such as electric and magnetic dipoles, quadrupoles, and so forth [61]. For specific sizes of particles, electric and magnetic dipoles may be excited simultaneously and their radiations may interfere differently in the backward and forward directions, hence, producing directional scattering – a phenomenon also known as Kerker scattering [62]. Hinamoto et al. [63] recently demonstrated the use of Fourier imaging to spectrally resolve radiation patterns of a single subwavelength Mie resonator. They were able to detect a suppressed backward scattering due to Kerker scattering as well as to selectively measure the contributions of electric and magnetic dipoles via a polarization-resolved Fourier imaging technique [63].

If we now consider elongated nanoparticles such as rectangular nanoantennas, Shegai et al. [48] used the BFP imaging method to analyze radiation patterns of long silver nanowires [48], by blocking light from the excitation source using an iris in the image plane of the microscope. With that configuration, they were able to demonstrate highly unidirectional broadband emission from the nanowires, hence, showing that the longitudinal shape of the object sets a preferential direction for light radiation (see Figure 2h).

The aforementioned experiments only enabled imaging a fraction of the total BFP. In order to fully filter out the unwanted light from the illumination source, Sersic, Tuambilangana, and Koenderink [47] exploited a dark-field microscopy configuration in which the objects under study are excited through a prism in total internal reflection mode. In that configuration, all of the incident laser is reflected while the radiation of the excited nanostructure is detected in transmission. One can, therefore, collect meaningful scattered signal at all angles in the full BFP region. Thanks to that technique, as displayed in Figure 2(g), the authors were able to analyze full radiation patterns of single Au nanowires with their observations complemented with an analytical framework based on diffraction theory. These experiments helped understand the way light is scattered from nanowires, in which the radiation pattern is similar to that of a point-dipole multiplied by a sinc^2^ function along the plane transverse to the wire [47]. These different works experimentally demonstrate that the shape of the scatterer imposes a directionality to the radiated field.

Shegai et al. [64] also provided another interesting method to avoid the illumination issue by analyzing the Raman signals from individual dimer and trimer gold nanoparticles decorated with Rhodamine-6G dye molecules. A combination of long-pass filter and dichroic mirrors was used to discriminate the Raman signal from the source and to image the full BFP. The study allowed resolving the 3D orientation and symmetry of the nanoantennas as well as the interaction and coupling of nanoparticles in dimers and trimers [64]. Using a similar strategy based on Raman scattering, Zhu et al. directly measured the so-called beamed Raman scattering using several plasmonic antennas and measuring the radiation patterns in the BFP [65]. By using the different angular distributions of the Raman and fluorescence signals from a film-coupled nanowire cavity, Vasista et al. pointed out an interesting technique to discriminate overlapping molecular emission processes [66]. More recently, Bag et al. used structured light and complex multipolar interferences in silicon nanoparticles to produce Kerker effects and enable precise nanoparticle localization via Fourier imaging [67].

### 3.3 Emitters coupled to scatterers: tailoring radiation patterns

We have seen previously in this section that Fourier imaging provides an elegant method to directly measure the angular-distribution of light arising from emitters and scatterers as well as to derive fundamental aspects of these radiators, such as their spatial orientation and multipolar nature. In the following, we describe how engineered environments can be used to redirect light emission from emitters and the role played by Fourier imaging to understand the emitter-nanostructure coupling.

As explained previously, dipole emitters radiate into well-defined directions – the “doughnut” pattern – set by the orientation of the dipole. Unfortunately this radiation pattern often reduces collection efficiency in nanophotonic devices. Therefore, a desirable goal is to redirect light toward tailored directions, something which may be accomplished by adding an intermediary between the emitter and the farfield, an optical antenna. In 2008, Taminiau et al. showed how optical antennas can redirect and amplify light emission from single molecules [68]. To excite the emitters and probe their emission, they leveraged a scanning probe microscope coupled with an aperture near-field probe onto which an aluminum nanoantenna was sculpted. By scanning the antenna over a sample containing randomly placed single emitters, the authors were able to reversibly trigger near-field coupling between the emitters and the antenna. As half-wavelength antennas are much more efficient energy radiators than point-dipoles, the total detected emission from the coupled emitter-antenna system is dominated by that of the antenna. As a result, the antenna redirects light emission from the source. Although the authors did not measure the angular distribution of light emission, they conducted numerical simulations of what would be the light intensity in the BFP [68] and concluded that Fourier imaging would have provided clear and distinct signatures of emission patterns for coupled and uncoupled molecules.

**Figure 3:**
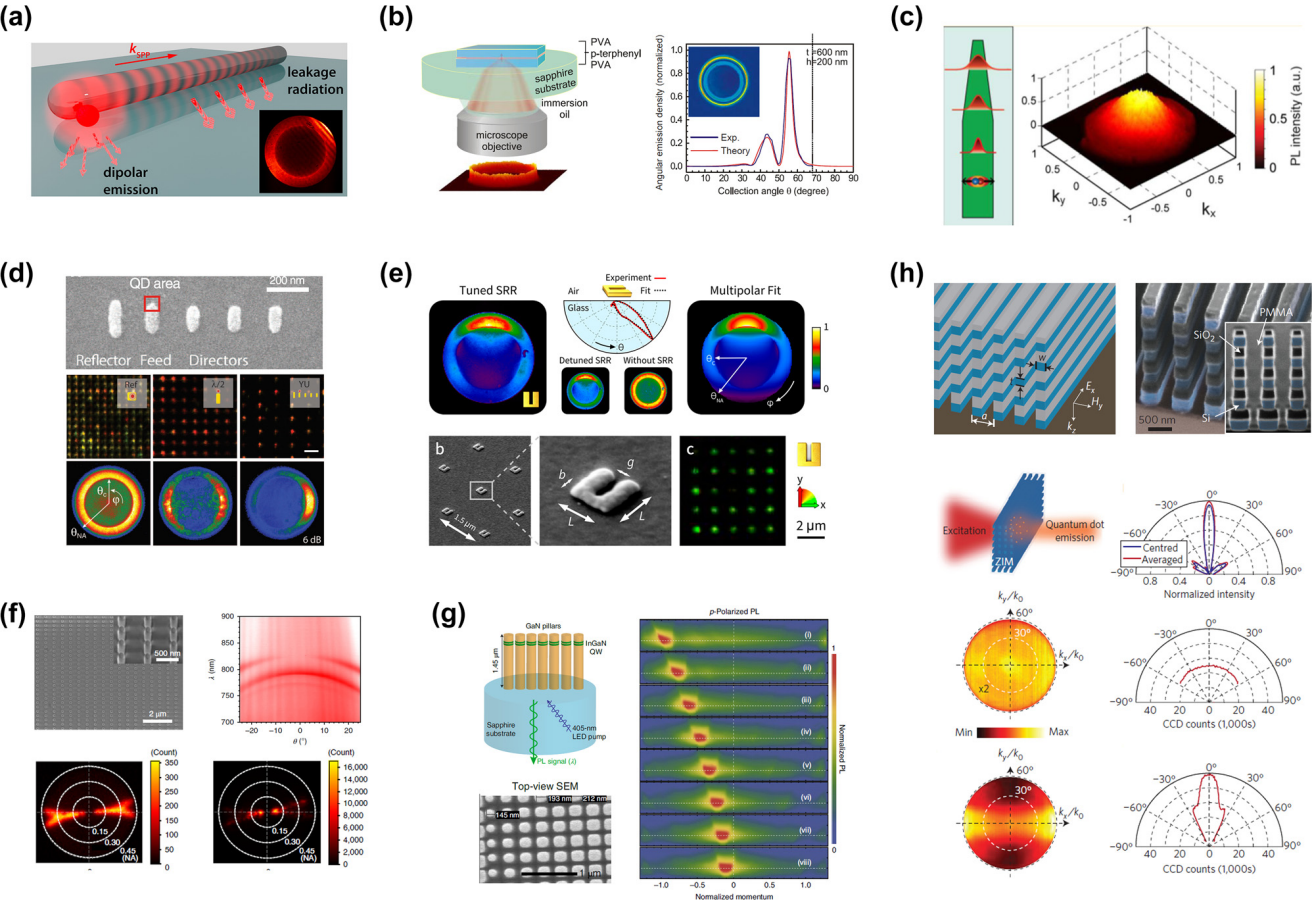
Various means of controlling the directionality of light emitters and their corresponding angular distribution of light emission measured by Fourier imaging. (a) Fourier imaging enabled understanding the position dependent coupling and radiation channels of rare-earth doped emitters coupled to a nanoantenna [69]. (b) Planar dielectric antenna containing a single emitter enabling near-unity collection efficiency of single-photon emission [70]. As shown by Fourier imaging, all of the light is funneled into a circularly shaped pattern emitted in the far-field at large angles. (c) A quantum dot inside a III–V nanowire with tailored diameters forming an optical waveguide with directional vertical emission, as imaged in the BFP [71]. (d) Directional emission of quantum dots coupled to half-wave and Yagi-Uda antennas [74]. (e) Split-ring resonators (SRRs) supporting multipolar resonances, which interfere in the far-field, hence, producing highly directional emission in a large bandwidth, as measured in the Fourier space [75]. (f) GaAs nanopillar supporting a bound state in the continuum (BIC) at normal incidence [76]. An array of these GaAs antennas produce a lasing action with high vertical directionality, as confirmed by BFP imaging. (g) Asymmetric incoherent light emission is produced at tailored angles by spatially arranging InGaN/GaN quantum well nanopillars of different widths on a substrate [77]. (h) Incoherent isotropic emitters in a zero-index medium emit coherently in the direction normal to the surface [78]. (a) Reproduced with permission [69]. Copyright 2013, American Chemical Society. (b) Reproduced with permission [70]. Copyright 2011, Nature Publishing Group. (c) Reproduced with permission [71]. Copyright 2014, American Chemical Society. (d) Reproduced with permission [74]. Copyright 2010, American Association for the Advancement of Science. (e) Reproduced with permission [75]. Copyright 2013, Nature Publishing Group. (f) Reproduced with permission [76]. Copyright 2018, Nature Publishing Group. (g) Reproduced with permission [77]. Copyright 2020, Nature Publishing Group. (h) Reproduced with permission [78]. Copyright 2013, Nature Publishing Group.

As the precise localization of the emitter with respect to the coupled nanostructure plays an important role in the overall properties of the system, Hartman et al. used Fourier imaging to analyze the influence in the radiation patterns of the relative distance between rare earth ion-doped nanocrystals and a plasmonic nanowire [69] (see Figure 3a). In the spirit of enhancing light collection, Lee et al. designed a planar dielectric antenna containing a single emitter and demonstrated a near-unity collection efficiency of single-photon emission. In this configuration, as shown by Fourier imaging, all of the light is funneled into a circularly shaped pattern emitted in the far-field at large angles (see Figure 3b) [70]. Another approach used to produce directional emission and large collection efficiencies consists in shaping the emitters as antennas. Bulgarini et al. introduced a semiconductor quantum dot inside a III–V nanowire with tailored diameters so as to form an optical waveguide with directional vertical emission (Figure 3c), hence, enabling a very high collection efficiency by a single mode fiber [71]. Jaffal et al. used a similar approach to demonstrate single-photon emission from needle-like InAs/InP QDs-nanowires systems [72]. In both these works, the vertical directionality of emission was directly demonstrated using Fourier imaging. In the same spirit but with a different geometry, Khoury et al. exploited Mie resonances in silicon resonators to increase the vertical extraction efficiency of G centers at telecom wavelengths toward an optimized coupling with optical fibers [73].

A more complex means of shaping emission direction consists in exploiting engineered nanoantennas. Curto et al. demonstrated that BFP-imaging can indeed provide direct evidence of directional emission from quantum dots coupled to nanoantennas [74]. By placing single core–shell QDs at close proximity to different integrated gold nanoantennas (half-wave dipole and Yagi-Uda), it was shown that radiation patterns, which before this had azimuthal symmetries, could be transformed to be bi-directional when coupled to half-wave dipole antennas or unidirectional when coupled to Yagi-Uda antennas (see Figure 3d) [74]. More recently, Ho et al. used a similar Yagi-Uda antenna (albeit silicon based) coupled to a gold bowtie nanoantenna and demonstrated highly directional light emission from the gold antenna [79]. A similar Fourier imaging approach was used by Peter et al. to demonstrate unidirectional emission from QDs coupled to dielectric antennas [80].

Having direct access to the radiation pattern of emitter-nanoantenna systems helped to further expand the directional control of light emission through the design of more complex antennas supporting multipolar resonances [75], [81]. In tailored split-ring resonators (SRRs), the scattering patterns of electric dipole, magnetic dipole, and electric quadrupole resonances interfere in the far-field to produce highly directional emission in a large bandwidth. This concept was demonstrated by measuring the directional emission originating from QDs coupled to SRRs using Fourier imaging techniques Figure 3e.

## 4 From coupled scatterers to nonlocal metasurfaces

Up to this point, it is now clear that Fourier imaging is highly useful for measuring the radiation patterns of single or coupled scatterers. It becomes even more versatile for studying resonant effects from ordered scatterers or periodic photonic structures such as photonic crystals or resonant metasurface periodic lattices (i.e., nonlocal metasurfaces) [82]–[86]. For example, the tight localization, Δ*x*, in real space of small single scatterers leads to a broad distribution in momentum space, Δ*k*, for photonic modes, as imposed by the Fourier transform relation Δ*x*Δ*k* ∼ 1. However, when scatterers are orderly arranged, interferential effects can be harnessed to engineer highly directional scatterings, which would be experimentally evidenced through Fourier imaging. Another illustrative example is dealing with Bloch mode resonances in a subwavelength lattice of a photonic crystal or resonant metasurface. These resonances are delocalized in real space but are generally tightly localized in momentum space.

In this section, we will first review a few illustrative works where Fourier imaging has been used to evidence the interference effect of far-field emission from coupled resonators designed to tailor light emission direction across a broad range of angles, from horizontal to vertical emission. Then, we will delve into more details and examples of how Fourier imaging of far-field emission can be combined with other optical elements for spectrally, polarization-, or phase-resolved measurements, serving as a powerful tool to map different physical quantities of Bloch resonances in momentum space.

Ha et al. designed GaAs nano-pillars supporting a dipolar resonance to produce a bound state in the continuum (BIC) at normal incidence. Using an array of these GaAs antennas, they demonstrate by Fourier imaging lasing action with high vertical directionality as governed by the high quality factor at k-points near the edges of the BIC (see Figure 3f) [76]. Using a similar arrangement, Hoang et al. designed chains of coupled Mie resonators, which, this time, produce in-plane directional lasing in an integrated device [87]. In between vertical and horizontal emission, Iyer et al. demonstrated, through Fourier imaging, how asymmetric incoherent light emission can be produced at tailored angles by spatially arranging InGaN/GaN quantum well nanopillars of different widths on a substrate Figure 3g [77]. The same principle was used by Khaidarov et al. to demonstrate beam deflecting LEDs [88].

Another type of farfield direction control was demonstrated by Moitra et al., using all-dielectric zero index metamaterials (ZIM) [78]. By exploiting a Dirac dispersion at the Γ point, both the refractive index and the spatial phase change are near-zero. As a result, incoherent isotropic emitters in such a ZIM medium emit coherently in the direction normal to the surface. This effect was directly demonstrated by Fourier-imaging light emission from PbS QDs integrated in a vertically stacked silicon rods metamaterial structure (see Figure 3h) [78].

Another nice example is the work of Bleckmann et al. in which the authors have studied the coupling between plasmonic waveguide array ranged in a bi-particle lattice [89]. The coupling between nearest neighbor waveguides in the design emulates the Su–Schrieffer–Heeger (SSH) model of 1D topological insulator [90], and the photonic lattice exhibits the bulk material of the insulator in SSH model. Fourier imaging has been used to evidence a localized topological edge state at the interface between two photonic insulators made of trivial and nontrivial topology.

### 4.1 Measuring the energy-momentum dispersion

Most of the examples discussed in previous sections are intensity mappings of scattering/emitting light in momentum space without resolving the emission in terms of energy/wavelength. In these examples, resolving the dependence of light emission along the energy axis is hindered by the broadband operation of the optical antenna or is sufficiently narrow so as to make trivial the wavelength dependence. However, the interplay between energy and momentum is at the heart of resonant dielectric/plasmonic periodic structures since the photonic energy-momentum dispersion dictates the propagation, emission, absorption mechanism, as well as the topological phase of light-waves in nanostructures [91].

**Figure 4:**
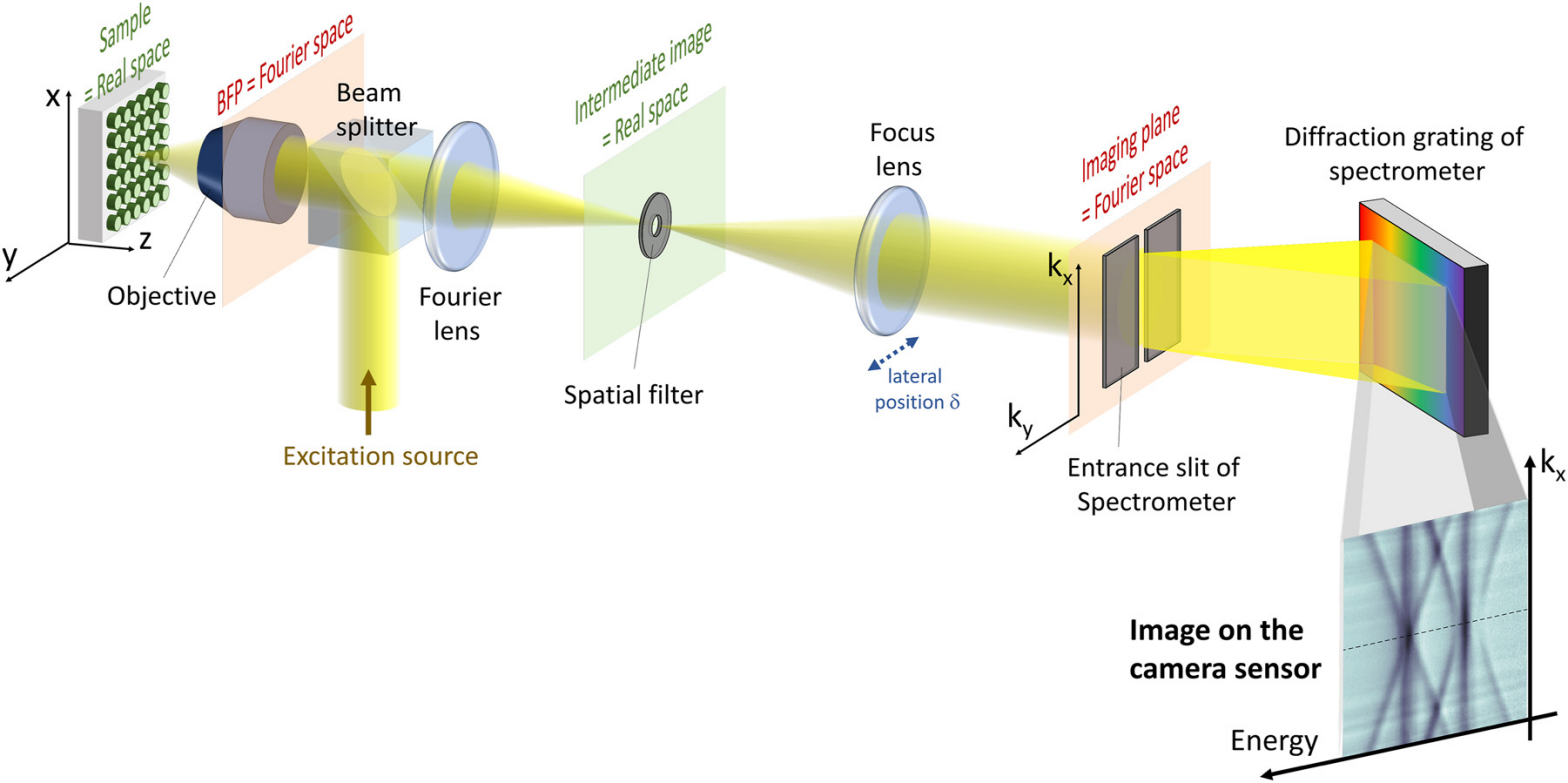
Scheme of a typical setup for measuring the energy-momentum dispersion characteristics of in reflected signal from a nanostructured sample. The sample is excited by a focused incident beam. The scattered signal is collected through the same microscope objective as the excitation. The back-focal plane (BFP) of the objective, corresponding to the Fourier space, is projected into the imaging plane by a set of two lenses: a Fourier lens and a focusing lens. A spatial filter can be eventually implemented on the intermediate image of the sample. The imaging plane is positioned at the entrance slit of a spectrometer that selects a given value of *k*_*y*_ = *k*_0_. The value of *k*_0_ is finely tuned by shifting the imaging plane with respect to the slit. This can be done, for example, by changing the lateral position *δ* of the focusing lens. A camera sensor is positioned at the output plane of the spectrometer. The image recorded by the sensor has two axes corresponding to energy (or wavelength) and *k*_*x*_, respectively. This setup can be adapted to study emission or transmission signals. More sophisticated versions of the setup include polarization elements in the excitation/collection path or implementing a spatial filter for the excitation.

In the same fashion as electronic band structures of semiconductors, photonic band structures are usually presented along an in-plane momentum direction, for example *k*_*x*_, with its perpendicular component *k*_*y*_, both of them composing the transverse momentum vector $k_{\|}=k_{x}{\hat{u}}_{x}+k_{y}{\hat{u}}_{y}$ . Figure 4 presents a typical setup to obtain the dispersion characteristic *E*(*k*_*x*_) of a nanostructured sample at a given *k*_*y*_ = *k*_0_ in a single shot measurement. In this scheme, the back focal plane is imaged onto the entrance of an imaging spectrograph with *k*_*x*_ oriented parallel to the entrance slit, and then the grating inside the spectrograph disperses the incoming signal along the energy axis. Finally the resulting image is captured in the camera sensor located at the output plane of the spectrograph. From the recorded image, the dispersion curves are revealed in the dips/peaks in reflectivity [92], [93]–[96], transmission [76], [97], [98], or photoluminescence [98]–[101].

**Figure 5:**
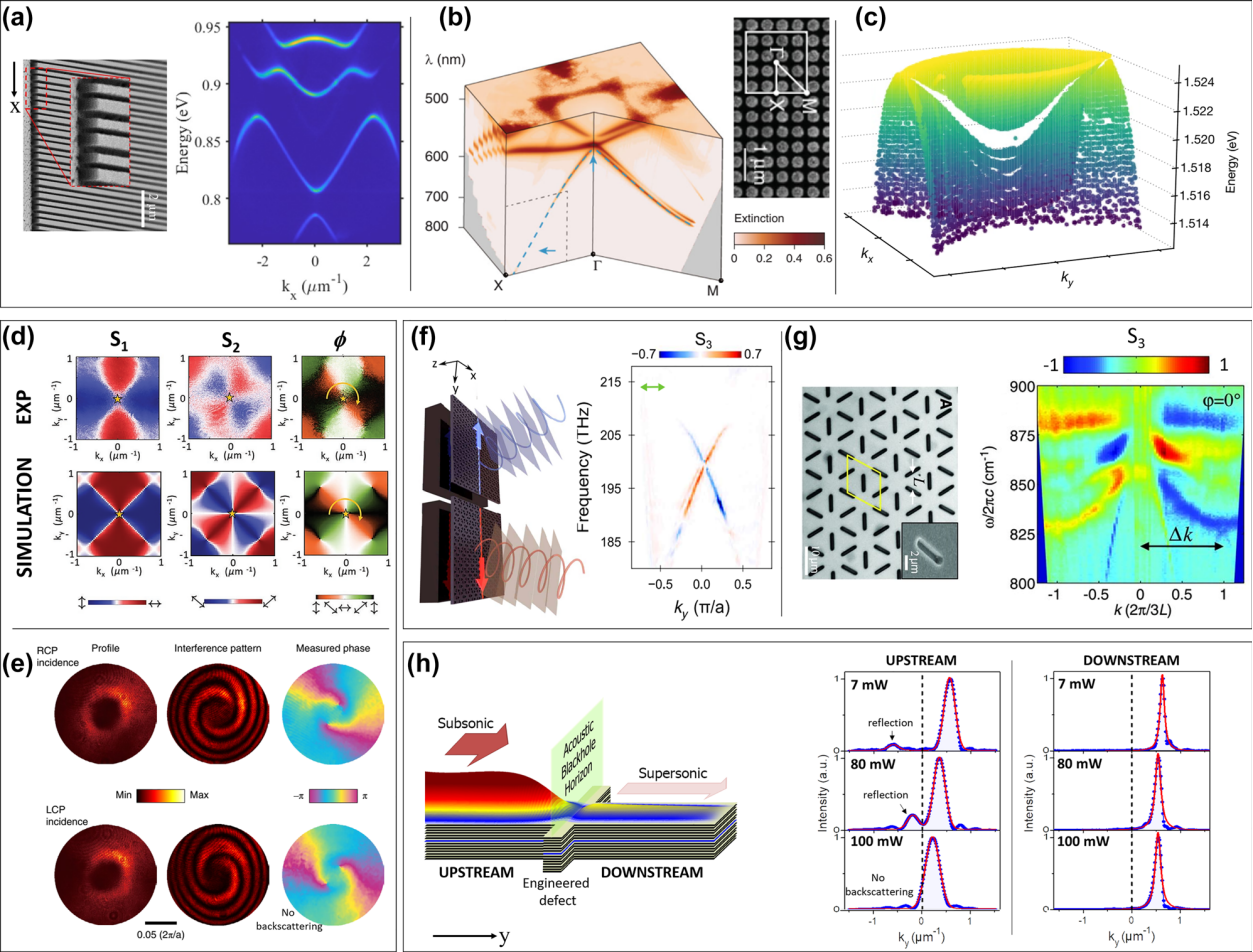
Extension of Fourier imaging, towards resolving all aspects of light. (a) Energy momentum dispersion of light emission from a 1D silicon-based metasurface, adapted from Ref. [99]. (b) Tomographic reconstruction of the dispersion cut slices from a 2D polymer-based photonic crystal, adapted from Ref. [92]. (c) Tomographic reconstruction of the dispersion surface of a GaAs grating, adapted from Ref. [102]. (d) Demonstration of a polarization vortex from a polariton BIC (adapted from Ref. [103]). The top panels are experimental results at different applied voltages. The lower panels are the corresponding theoretical calculations. (e) Demonstration of phase vortices, which are pinned at optical BIC (adapted from Ref. [104]). (f) Observation of counter propagating edge-states of opposite pseudo-spin in photonic topological valley Hall effect (adapted from Ref. [105]. (g) Experimental demonstration of photonic Rashba effect with metamaterial made from artificial kagome lattice of micro antenna (adapted from Ref. [106]). (h) Combination of real space and momentum space mapping to demonstrate the formation of a sonic black hole when a quantum fluid of light flowing across an engineered defect (adapted from Ref. [107]). The Fourier imaging after spatial filtering provide the wavevector peak of light propagation in the upstream and downstream region, from which the flow speed can be extracted. (a) Reproduced with permission [99]. Copyright 2019, IEEE. (b) Reproduced with permission [92]. Copyright 2018, American Physical Society. (c) Reproduced with permission [102]. Copyright 2022, Nature Publishing Group. (d) Reproduced with permission [103]. Copyright 2021, Wiley-VCH. (e) Reproduced with permission [104]. Copyright 2020, Nature Publishing Group. (f) Reproduced with permission [105]. Copyright 2020, American Association for the Advancement of Science. (g) Reproduced with permission [106]. Copyright 2013, American Association for the Advancement of Science. (h) Reproduced with permission [107]. Copyright 2015, American Physical Society.

One of the simplest objects to study is the case of a 1D periodic lattice, for which the energy-momentum dispersion is measured by aligning the entrance slit with the lattice direction. This configuration was used in a 1D metasurface to demonstrate a two orders of magnitude enhancement of photoluminescence emission from silicon (Figure 5a), which was attributed to the reshaping of the silicon emission by the band dispersion of the metasurface [99].

For a photonic crystal with a 2D periodic lattice, measuring the band structure along a single direction would only give a partial view of the device’s optical properties. The direction of the measured in-plane momentum can be selected by rotating the sample in the *xy* plane. As an illustration, Figure 5b depicts a 3D reconstruction of the band diagram, with tomographic slices of energy-momentum band structures, measured from a polymer-based photonic crystal with a metal substrate, as reported by Zhang et al. [92]. We note that the horizontal slices of the tomography reconstruction, corresponding to isofrequency images, can alternatively be obtained by replacing the spectrograph with a set of spectral filters [92], [108], [109] or a tunable monochromatic excitation [110].

Ultimately, it is possible to fully reconstruct the dispersion surface *E*(*k*_*x*_, *k*_*y*_) of photonic resonances. This is achieved by extracting the dispersion relationship *E*(*k*_*x*_) for various values of *k*_0_ in *k*_*y*_. To do so, the sweeping of *k*_0_ can be performed by shifting the image of the back focal plane with respect to the entrance slit, for example, by tuning the lateral position *δ* of the focusing lens (see Figure 4). As an illustration, Figure 5c presents the dispersion surface of polaritonic modes in a GaAs-based grating at cryogenic temperature, as reported by Ardizonne et al. [102]. Such a reconstruction leads to the demonstration of two polaritonic modes with distinctive characteristics: one exhibiting a local minimum while the other exhibits a saddle point at *k*_*x*_ = *k*_*y*_ = 0.

### 4.2 Mapping polarization and phase patterns

Other than its wavelength and momentum, light is also characterized by two other key parameters: polarization and phase. The polarization state of light is mathematically represented as a vector in the Poincaré sphere with the three Stokes parameters *S*_1_, *S*_2_, and *S*_3_ being its coordinates [111]. The two first Stokes parameters correspond to the linearly polarized component, thus indicating the orientation of light polarization, given by $\phi =0.5\arctan\left(S_{2}/S_{1}\right)$ . By mapping these two components in momentum space, polarization singularities can be visualized and may give signatures of topological charges such as those of bound states in the continuum (BIC) [94], [102]–[104], [112], [113]. The third component *S*_3_ corresponds to the circularly polarized component and is usually referred to as the photonic pseudo-spin (or spin angular momentum of light) [114]. By mapping polarization patterns in momentum space, one may reveal the pseudo spin texture of the optical spin Hall effect [115–119]. Technically, mapping the Stokes parameters in momentum space is obtained by processing BFP images taken with different sets of polarization elements (half-wave plate, quarter-wave plate and polarizer) [120]. An example of such mapping is depicted in Figure 5d, adapted from Ref. [103], in which the topological nature of polariton BICs is revealed by the observation of polarization singularity (i.e., vortex in the pattern of *ϕ*(*k*_*x*_, *k*_*y*_). In this study, the pattern of polarization orientation *ϕ*(*k*_*x*_, *k*_*y*_) is obtained from the measurements of the Stokes parameters *S*_1_(*k*_*x*_, *k*_*y*_) and *S*_2_(*k*_*x*_, *k*_*y*_).

Concerning the phase of light fields, the phase information in momentum space can be obtained via interference measurements where the signal is interfered with a reference beam. An example of such experiment is depicted in Figure 5e, adapted from Ref. [104], showing phase vortices in momentum space of light transmitted through a photonic BIC.

Moreover, certain photonic effects are only manifested when mapping more than one parameter in momentum space. A distinctive case is the photonic spin-dependent energy-momentum dispersion, which is the trademark of novel photonic effects such as the topological photonic valley Hall effect, which results in counter propagating edge states [105], [121]. Figure 5f, adapted from the work of Parappurath et al. [105], depicts the experimental evidence of this effect in a silicon photonic crystal. Here, the observation of two propagating states of opposite group velocity and opposite photonic pseudo-spin (i.e., *S*_3_) evidences clearly the photonic valley Hall effect. Another effect that is manifest in the spin-dependent energy-momentum dispersion is the optical analog of the Rashba effect as induced by photonic spin–orbit coupling [106], [122]. Figure 5g, adapted from the work of Shirit et al. [106], presents the demonstration of such effect using metamaterial made from an artificial kagome lattice of micro antenna. Here, photonic spin–orbit coupling is revealed by the splitting of the photonic bands having opposite spins.

### 4.3 Combining measurement from real and momentum space

Finally, what happens if imaging in momentum space is insufficient? A direct case involves the problematic overlap between the resonant scattering signal and specular reflection at the excitation spot in a reflectivity experiment on a photonic crystal. One strategy to eliminate the specular signal is to perform a resonant Rayleigh scattering measurement via cross-polarization imaging at the expense of distinguishing between modes of opposite polarization [103], [123]. Another strategy, without losing polarization resolution, is to spatially decouple the specular signal and the scattering signal by performing the measurement in momentum space away from the excitation spot. This can be achieved by using an optical system with an intermediate image plane, allowing for the blocking of the excitation spot using a spatial filter. A good example of this technique is the result previously mentioned in Figure 5f from Ref. [105]. This technique requires a combination of analysis in both momentum space, for the imaging, and real space, for the spatial filtering.

Another illustrative example that may require a combination of real space and momentum space resolution is the study of light propagation in the presence of a potential landscape or/and a strong nonlinearity medium [107], [124], [125]. In such a configuration, the light propagation velocity, extracted from the energy-momentum dispersion, would depend on the spatial coordinate. One may probe such dependence, i.e., velocity profile, by performing the Fourier image when selecting different parts of the light flow thanks to a spatial filter put at the intermediate image plane. The main difference from this experiment to the one that gets rid of the specular reflection, previously discussed, is that the spatial filter in the previous case is for filtering out a small portion of the real space image, while the one in this experiment is for selecting a small part of the real space image. That leads to a trade-off between spatial resolution and momentum resolution: the more information you gather on light location, the more you lose on light momentum. A perfect illustration of a combination between real space and momentum space imaging is shown in Figure 5h, adapted from Ref. [107]. In this experiment, the authors have demonstrated a photonic analog of black hole by studying a nonlinear photonic flow propagating across an engineered defect that divides the flow into upstream and downstream region. The Fourier image with spatial filtering provides the light propagation velocity given by the wavevector peak, in the upstream and downstream. Comparing these velocities to the speed of sound in the same region would demonstrate the subsonic/supersonic nature of the upstream/downstream region. As a consequence, the downstream (supersonic) region corresponds to an acoustic blackhole, which is separated from the upstream (subsonic) region by a sharp horizon.

The above examples illustrate nicely how Fourier imaging in the far-field, when synergistically combined with advanced optical tools such as high-resolution spectrometers, polarization mapping techniques, and phase measurement from interferometers, has emerged as a remarkably powerful tool in the study of photonic phenomena. This combination unlocks a vast realm of possibilities, ranging from exploring fundamental problems in areas such as topological physics and analog gravity, to driving innovations in practical applications like lasers, light-emitting devices, and optical trapping. The versatility and depth of analysis provided by this integrated approach not only deepen our understanding of complex photonic behaviors but also pave the way for pioneering advancements in both theoretical research and technological developments.

## 5 Summary and future perspectives

We have shown in this review how a converging lens presents the faculty of computing the Fourier transform of an incoming light beam. This natural property of lenses provides a strikingly simple means to measure the angular distribution of light emission, by imaging the BFP of the lens. As described in this review, having a direct measurement of the projected angular distribution of light grants an invaluable access to light emission/scattering processes as well as their interplay with the surrounding environments. Throughout this Review, we described how Fourier imaging already played a significant role in understanding the fundamental mechanisms governing light emission and scattering, but also in optimizing systems of nanostructures, from emitter-nanoantennas systems to collective effects such as the band structures of metasurfaces and photonic crystals. In that regard, we can draw a parallel between these on-going developments and those, more mature of the angle-resolved photoemission spectroscopy (ARPES) technique, which plays a significant role in understanding electronic structures in solid-state physics [126].

In the following, we review different exploration avenues for future research and developments of the technique.

### 5.1 Instrumentation & resolution

Interestingly, the optics used in most Fourier imaging setups are optimized for real-space measurements but not for BFP imaging [26]. Future improvements of setups using custom-made lenses and objectives for the Fourier transform would, therefore, definitely help to enhance typical resolutions.

Other potential improvements could be done to provide direct measurements of the complete three-dimensional band diagram of nanophotonic devices. As explained in Section 4, two straightforward implementations consist in sequentially recording either the isofrequency contours (i.e., one wavelength at a time, using tunable light source or tunable filters) or by recording slices of the momentum space (by shifting the image of the BFP with respect to the spectrometer’s slit). Isofrequency contours or momentum slices can later on be “stacked” and reconstructed as 3D band diagrams [92].

Another recently proposed and demonstrated technique is the k-space hyperspectral birefringent common-path interferometer setup [127]. In such a setup, the light collected at the BFP of an objective is directed to an interferometer that splits the light field in two replicas with a controlled delay. Once recombined and plotted as a function of the delay, these replicas form an interferogram. As explained in Section 2, the Fourier transform of such an interferogram yields a frequency spectrum. The combination of BFP imaging and interferogram, hence, grants access to the full angular and momentum dispersion of light in a single measurement. Interestingly, this method combines the two different aspects of Fourier transforms: the time-frequency Fourier transform (transforming an interferogram into a frequency spectrum) and the decomposition of a light field into its wavevector components (spatial Fourier transform). In their work, Genco et al. [127] exploit the so-called “Translating-Wedge-based Identical pulses eNcoding System (TWINS)” in place of more cumbersome solutions such as Mach–Zehnder or Michelson interferometer, hence, improving stability and footprint while strongly reducing the time needed to fully reconstruct the full 3D bandstructure of a sample.

### 5.2 Single-shot multiple analysis

Given the simplicity of Fourier imaging – most of the time one simply needs to add a single lens in their optical setups – it can easily be combined with other techniques toward resolving energy, polarization, phase, and so on, as described in the previous section. We thus foresee a generalized use of various metasurfaces as custom-made optics in Fourier setups, to either enhance the width of angular view [128] or to add functionalities such as decoupling amplitude and polarization [129]. By combining Fourier optics with metasurfaces enabling single shot polarization analysis [130], quantitative phase imaging [131], orbital angular momentum resolution [132], [133], 4D imaging [134], or spectroscopic ellipsometry [135], compact setups could be designed to fully resolve all aspects of light states while still keeping its “instantaneous” character.

### 5.3 Dynamic measurements

Compared to other alternative methods, such as the mechanical scanning of detectors, Fourier imaging is not only simpler but also much faster, as all angles are measured simultaneously and instantaneously. Only a few studies actually made use of such a real time measurement [28], [136], but we foresee that dynamic Fourier imaging should open new directions of research for real-time imaging of biological systems or living organisms [137] as well as in tunable and reconfigurable devices [138]. Given the potential complexity of data sorting and analysis in such dynamic k-space measurements, the use of artificial intelligence algorithm for sorting data and reconstructing patterns would be of great help.

### 5.4 Feedback between real and k-space

The instantaneous character of Fourier measurements could also be used for post-trimming or fine-tuning of devices by setting up a feedback loop between real space and k-space. If one has a direct possibility to modulate the samples properties, using, e.g., electrodes, thermal actuation, or lasers, the effects of adjustments made in the real space could be monitored in the Fourier space, toward specific radiation patterns or dispersion in the energy-momentum space. Further extending this idea, future years should see the development of nanophotonic devices designed in the momentum space to produce specific angular distribution and whose fabrication patterns are generated through their Fourier transform [139]. We can even imagine using laser projections of desired patterns in the Fourier space that are re-injected in the real space to expose photosensitive materials such as photoresist or phase-change materials, hence, producing tailor-made devices. Such a process bears resemblance with the computational methods used to design metasurfaces: iterative methods such as the Gerchberg–Saxton algorithm are used to design metasurfaces starting from a target image that is Fourier-transformed multiple times to produce a phase mask in the real space.

To continue with the analogy with computing, as Fourier transforms were first developed as mathematical tools, the field of Fourier optics should definitely see interesting outcomes in optical analog computing, computer vision, diffractive deep neural networks, and optical integrators [140]–[143].

To conclude, we believe Fourier imaging has already been playing a major role in the field of nanophotonics (as well as in other fields) and we foresee that the combination of simplicity, compactness, and rapidity will make it a widespread technique that could be used even in nonspecialists labs or industries. Furthermore, the fast evolutions in the field of metasurfaces should add exceptional possibilities to this technique, toward fast and compact setups enabling direct and simultaneous measurements of all aspects of light: energy, momentum, polarization, and phase.
